# Prevalence of metabolic syndrome among patients with hepatocellular carcinoma of different etiologies: a retrospective study

**DOI:** 10.1186/s13027-024-00575-6

**Published:** 2024-05-01

**Authors:** Da-Long Yang, Shao-Ping Liu, Hong-Liang Wang, Jian-Rong Li, Jia-Yong Su, Min-Jun Li, Yu-Xian Teng, Zhu-Jian Deng, Zhong-Hai Li, Jian-Li Huang, Ping-Ping Guo, Liang Ma, Zhen-Zhen Li, Jian-Hong Zhong

**Affiliations:** 1 Hepatobiliary Surgery Department, Guangxi Liver Cancer Diagnosis and Treatment Engineering and Technology Research Center, Guangxi Medical University Cancer Hospital, Nanning, China; 2 Hepatobiliary Surgery Department, The Eighth Affiliated Hospital of Guangxi Medical University, Guigang, China; 3 Organ Transplantation Department, 923th Hospital of PLA Joint Logistic Support Force, Nanning, China; 4 Pathology Department, Guangxi Medical University Cancer Hospital, He Di Rd 71, 530021 Nanning, China; 5 Key Laboratory of Early Prevention and Treatment for Regional High Frequency Tumor (Guangxi Medical University), Ministry of Education, Nanning, China; 6 Guangxi Key Laboratory of Early Prevention and Treatment for Regional High Frequency Tumor Ministry of Education, Nanning, China

**Keywords:** Metabolic dysfunction-related fatty liver disease, Chronic hepatitis B, Hepatocellular carcinoma, Metabolic syndrome

## Abstract

**Aims:**

This study compared the prevalences of metabolic syndrome and of cardiac or kidney comorbidities among patients with hepatocellular carcinoma (HCC) associated with metabolic dysfunction-related fatty liver disease (MAFLD), chronic infection with hepatitis B or C virus (HBV or HCV), or the combination of MAFLD and chronic HBV infection.

**Methods:**

Medical records were retrospectively analyzed for patients with HCC who underwent hepatectomy between March 2013 and March 2023. Patients with HCC of different etiologies were compared in terms of their clinicodemographic characteristics and laboratory data before surgery.

**Results:**

Of the 2422 patients, 1,822 (75.2%) were chronically infected with HBV without MAFLD and HCV, 415 (17.2%) had concurrent MAFLD and chronic HBV infection but no HCV infection, 121 (5.0%) had MAFLD without hepatitis virus infection, and 64 (2.6%) were chronically infected with HCV in the presence or absence of MAFLD and HBV infection. Compared to patients chronically infected with HBV without MAFLD and HCV, those with MAFLD but no hepatitis virus infection showed significantly lower prevalence of cirrhosis, ascites, portal hypertension, alpha-fetoprotein concentration ≥ 400 ng/mL, tumor size > 5 cm, multinodular tumors and microvascular invasion. Conversely, they showed significantly higher prevalence of metabolic syndrome, hypertension, type 2 diabetes, abdominal obesity, history of cardiovascular disease, T-wave alterations, hypertriglyceridemia and hyperuricemia, as well as higher risk of arteriosclerotic cardiovascular disease. Compared to patients with MAFLD but no hepatitis virus infection, those with concurrent MAFLD and chronic infection with HBV showed significantly higher prevalence of cirrhosis, ascites and portal hypertension, but significantly lower prevalence of hypertension and history of cardiovascular disease. Compared to patients with other etiologies, those chronically infected with HCV in the presence or absence of MAFLD and HBV infection, showed significantly higher prevalence of cirrhosis, portal hypertension, ascites, and esophagogastric varices.

**Conclusion:**

Patients with HCC associated with MAFLD tend to have a background of less severe liver disease than those with HCC of other etiologies, but they may be more likely to suffer metabolic syndrome or comorbidities affecting the heart or kidneys.

## Introduction

The most frequent causes of hepatocellular carcinoma (HCC) are chronic infection with hepatitis B or C virus and alcoholic liver disease [1]. Nevertheless, the most frequent causes of mortality among HCC patients in recent decades have become non-alcoholic fatty liver disease and non-alcoholic steatohepatitis [2, 3]. Non-alcoholic fatty liver disease, which affects approximately 70% of overweight individuals [4, 5], has traditionally been defined as macrovesicular steatosis in at least 5% of hepatocytes of individuals consuming little or no alcohol who show no other identifiable cause of steatosis [6]. Recently, experts from 22 countries renamed non-alcoholic fatty liver disease as metabolic-associated fatty liver disease (MAFLD), which they defined as evidence of hepatic steatosis in the presence of overweight/obesity and/or type 2 diabetes and/or metabolic dysregulation [7, 8].

Having metabolic syndrome (MetS) increases one’s risk of developing MAFLD [9], which in turn increases risk of developing type 2 diabetes, cardiovascular disease and chronic kidney disease [10]. Nevertheless, patients with MAFLD-associated HCC experience less severe liver disease and higher rates of long-term survival after hepatectomy than patients chronically infected with hepatitis B virus in the presence or absence of MAFLD [11]. These considerations raise the question of whether the pre-hepatectomy presence of MetS and other comorbidities explains the different prognoses of patients with HCC related to MAFLD or viral hepatitis.

We explored this question by comparing the prevalence of MetS and other comorbidities among patients scheduled for hepatectomy to treat HCC associated with MAFLD, chronic infection with hepatitis B or C virus, or concurrent MAFLD and chronic infection with hepatitis B virus.

## Patients and methods

### Study design and population

Medical records were retrospectively examined for a consecutive series of HCC patients who underwent potentially curative hepatectomy between 11 March 2013 and 9 March 2023 at Guangxi Medical University Cancer Hospital (Nanning, China). Only preoperative clinicodemographic and laboratory data for these patients were extracted from the hospital’s central database. This study was approved by the Ethics Committee of Guangxi Medical University Cancer Hospital (approval LW2023160), which waived the requirement for written informed consent because of the retrospective study design.

To be included in the study, patients had to have biopsy-confirmed HCC involving MAFLD, chronic infection with hepatitis B or C virus, or concurrent MAFLD and chronic infection with hepatitis B virus; and they had to undergo hepatectomy. Patients were excluded if biopsies indicated intrahepatic cholangiocarcinoma (alone or concurrent with HCC), defined as adenoma, sarcoma, neuroendocrine tumor, or metastatic tumor; if they were diagnosed with HCC falling outside the etiologies mentioned above; if they had autoimmune hepatitis or drug-induced liver injury before hepatectomy; if they were taking drugs known to promote hepatic steatosis [12, 13]; or if their medical records were incomplete.

### Definitions of HCC etiologies and comorbidities

HCC was divided into one of four etiologies depending on whether it was associated with *(1)* MAFLD in the absence of chronic hepatitis virus infection, *(2)* chronic infection with hepatitis B virus in the absence of MAFLD or chronic infection with hepatitis C virus, *(3)* concurrent MAFLD and chronic infection with hepatitis B virus in the absence of chronic infection with hepatitis C virus, or *(4)* chronic infection with hepatitis C virus in the presence or absence of MAFLD and presence or absence of chronic infection with hepatitis B virus.

MAFLD was defined as the presence of hepatic steatosis through imaging or histology in addition to one of the following: overweight or obesity (defined as body mass index ≥ 23 kg/m^2^), type 2 diabetes, or evidence of metabolic dysregulation [7, 8]. Chronic infection with hepatitis B virus was defined as seropositivity for hepatitis B surface antigen, viral DNA, and/or antibodies against hepatitis B core protein. Chronic infection with hepatitis C virus was defined as seropositivity for antibodies against the virus.

Our central database did not record the data about waist circumference. Abdominal obesity was defined as body mass index ≥ 23 kg/m^2^ [7]. Type 2 diabetes was defined as fasting blood glucose ≥ 110 mg/dl, glycosylated hemoglobin ≥ 6.5%, history of diabetes, or the use of hypoglycemic medications or insulin [7]. Hypertriglyceridemia was defined as serum triglycerides ≥ 150 mg/dl [7], hyperuricemia, as a serum uric acid level > 420 µmol/L in men and > 360 µmol/L in women [14]; and hypertension, as three blood pressure measurements on different days indicating ≥ 130/≥85 mmHg, or history of hypertension, or current use of antihypertensives [7]. MetS is a condition that includes a cluster of risk factors specific for cardiovascular disease. The cluster of metabolic factors include abdominal obesity, high blood pressure, impaired fasting glucose, high triglyceride levels, and low HDL cholesterol levels. MetS was defined as abdominal obesity, hypertriglyceridemia, HDL cholesterol < 40 mg/dl for men or < 50 mg/dl for women, hypertension, and fasting glucose ≥ 110 mg/dl.

Cardiovascular disease was defined as coronary heart disease and stroke. Coronary heart disease was identified as myocardial infarction, coronary angiography, coronary stenting and/or coronary artery bypass surgery. Stroke was defined as intracerebral hemorrhage or cerebral infarction. Risk of arteriosclerotic cardiovascular disease was assessed using a scoring system as described [15], and “high risk” was defined as ≥ 10% on the scale. Chronic kidney disease was defined as an estimated glomerular filtration rate of < 60 ml/min per 1.73 m^2^, based on the CKD-EPI equation [16].

Hepatic steatosis, cirrhosis, ascites and portal hypertension were determined through analysis of imaging and/or histology. Esophagogastric varices were determined based on findings from esophagoscopy and gastroscopy. Premature atrial beats and T-wave alterations were determined from electrocardiographic reports. Abnormal plasma levels of creatine kinase, creatine kinase isoenzyme, or serum troponin I were defined based on our medical center’s respective normal ranges of 36–175 U/L, 0–24 U/L, or 0–28 µg/L. Patients declared whether they were current smokers or non-smokers, and current alcohol intake was defined as > 30 g/day for men or > 20 g/day for women [17].

### Statistical analyses

The Kolmogorov-Smirnov test was used to test normal distribution of continuous variables. Continuous data showing a normal distribution were reported as mean ± standard deviation, and inter-group differences were assessed for significance using one-way ANOVA with LSD post-hoc test. Continuous data showing skewed distribution were reported as median (quartile range), and inter-group differences were assessed using the Kruskal-Wallis test. The Bonferroni post hoc test was used for multiple comparisons when the Kruskal-Wallis test was significant. Categorical data were reported as n (%), and differences were assessed using Pearson’s chi-squared test. All statistical analyses were performed using GraphPad Prism 8.0 and SPSS 27.0 (IBM, Armonk, NY, USA). All statistical analyses with a *p* < 0.05 was considered statistically significant.

## Results

**Fig. 1:**
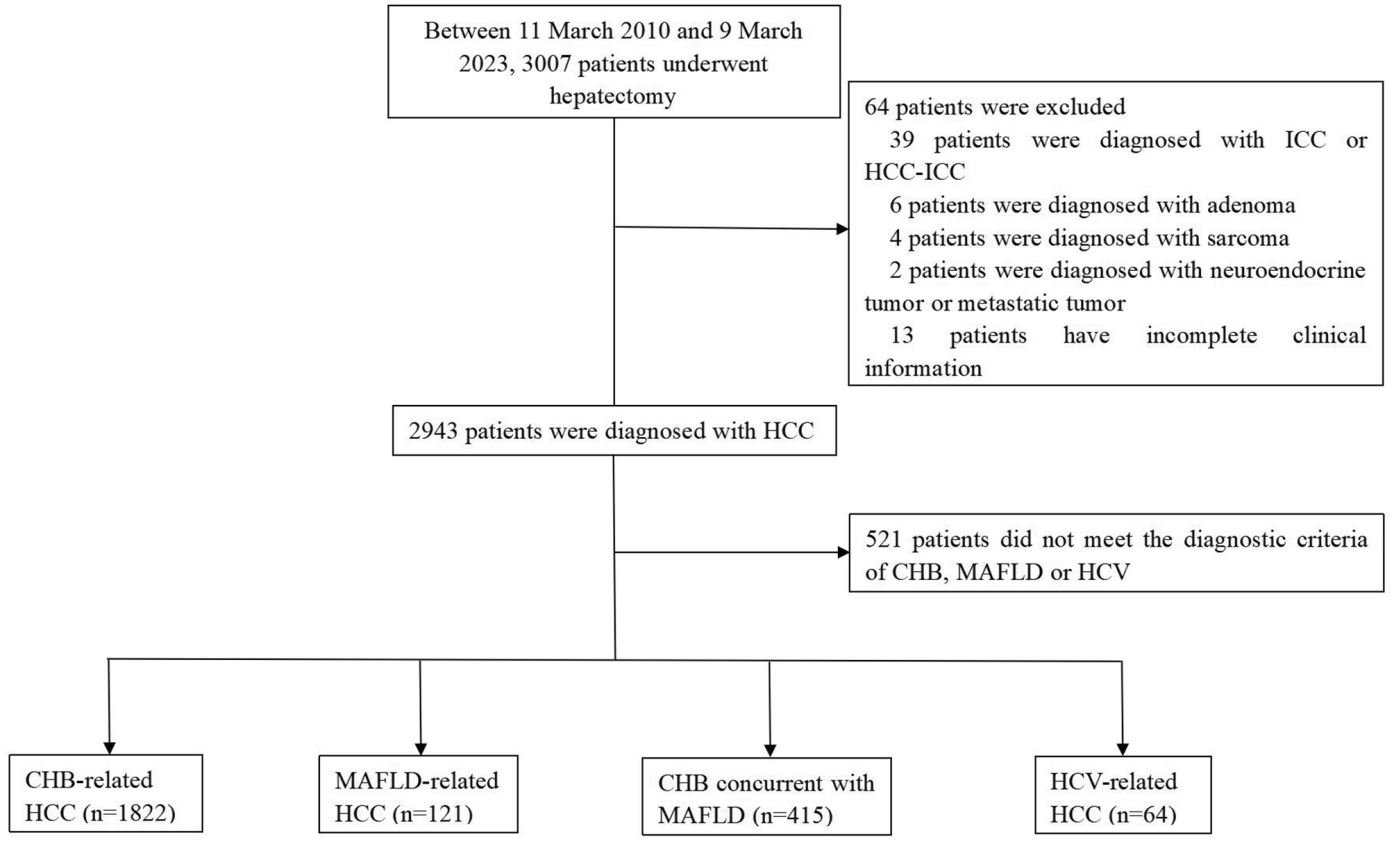
Flowchart of patient selection and stratification. HCC, hepatocellular carcinoma; ICC, intrahepatic cholangiocarcinoma; CHB, chronic infection with hepatitis B virus; HCV, chronic infection with hepatitis C virus; MAFLD, metabolic dysfunction-associated fatty liver disease

During the recruitment period, 3,007 patients underwent potentially curative hepatectomy at our medical center. We excluded 521 patients because they did not have MAFLD or chronic infection with hepatitis B or C virus, 51 because they had intrahepatic cholangiocarcinoma, and 13 because their medical records were incomplete. The remaining 2,422 patients were included in the final analysis (Table 1; Fig. 1).

**Table 1:** Clinicodemographic characteristics of hepatocellular carcinoma patients before potentially curative hepatectomy

Characteristic	Etiology of hepatocellular carcinoma						P
	CHB	MAFLD		CHB/MAFLD		HCV	
n	1822	121	415		64		
Age, yr	51.0 ± 11.0 ^a^	57.4 ± 11.0 ^a, b, c^	50.7 ± 10.2 ^b^		52.7 ± 11.2 ^c^		< 0.001^**^
Sex							
Male	1573 (86.3)	103 (85.1)	364 (87.7)		56 (87.5)		0.846^***^
Female	249 (13.7)	18 (14.9)	51 (12.3)		8 (12.5)		
Body mass index, kg/m^2^	22.4 ± 3.1 ^a, d^	24.5 ± 3.2 ^a, b^	24.6 ± 3.0 ^c, d^		22.5 ± 3.0 ^b, c^		< 0.001^**^
Current smoker	749 (41.1) ^a, b^	39 (32.2) ^a, c^	146 (35.2) ^b, d^		31 (48.4) ^c, d^		0.020^***^
Current alcohol drinker	621 (34.1)	49 (40.5)	141 (34.0)		23 (35.9)		0.538^***^
Blood pressure, mmHg							
Systolic	123.0 (112.0-134.0) ^a, b^	129.0 (118.0-142.0) ^a, c^	126.0 (119.0-138.0) ^b^		122.5 (110.3–137.0) ^c^		< 0.001^*^
Diastolic	79.5 (72.0–87.0) ^c, d^	83.0 (76.0–88.0) ^b, d^	82.0 (75.0–90.0) ^a, c^		79.0 (69.3–85.8) ^a, b^		< 0.001^*^
Fasting blood glucose, mmol/L	4.5 (4.1-5.0) ^a, b, c^	4.9 (4.5–5.8) ^c, d^	4.7 (4.3–5.4) ^b, d^		4.7 (4.2–5.5) ^a^		< 0.001^*^
Type 2 diabetes	159 (8.7) ^a^	24 (19.8) ^a^	62 (14.9) ^a^		11 (17.2) ^a^		< 0.001^***^
Hepatic steatosis	48 (2.6) ^a, b, c^	121 (100.0) ^a, d^	415 (100.0) ^b, e^		14 (21.9) ^c, d, e^		< 0.001^***^
Liver cirrhosis	1428 (78.4) ^a^	68 (56.2) ^a, b, c^	318 (76.6) ^b^		52 (81.3) ^c^		< 0.001^***^
Triglycerides, mmol/L	0.9 (0.7–1.2) ^a, b^	1.2 (0.8–1.7) ^a, c^	1.1 (0.8–1.5) ^b^		1.0 (0.8–1.5) ^c^		< 0.001^*^
Total bilirubin, µmol/L	13.9 (10.4–18.4)	14.5 (10.8–18.6)	13.8 (10.5–18.2)		13.1 (9.9–17.7)		0.728^*^
Alanine aminotransferase, U/L	37.0 (28.0–55.0) ^b, e^	32.0 (21.5–47.5) ^a, b, c^	37.0 (27.0–51.0) ^a, d^		53.5 (39.3–74.8) ^c, d, e^		< 0.001^*^
Aspartate aminotransferase, U/L	37.0 (27.0–54.0) ^a, c^	32.0 (26.0–45.0) ^a, b^	36.0 (29.0–50.0) ^b, d^		54.5 (34.0-76.5) ^c, d^		< 0.001^*^
Albumin, g/L	37.9 (35.1–40.7) ^a, d, e^	39.0 (36.3–41.1) ^c, e^	39.0 (36.3–41.9) ^b, d^		36.3 (32.9–40.1) ^a, b, c^		< 0.001^*^
Prealbumin, mg/L	184.8 ± 66.0 ^a, b, c^	219.1 ± 60.3 ^a, d, e^	205.9 ± 58.3 ^b, d, f^		157.1 ± 51.2 ^c, e, f^		< 0.001^**^
Urea nitrogen, mmol/L	4.9 (4.1–5.8)	5.1 (4.3-6.0)	4.8 (4.1–5.7)		4.7 (3.6–5.5)		0.113^**^
Prothrombin time, sec	12.6 (11.8–13.4) ^b, d^	12.0 (11.3–12.8) ^a, b, c^	12.4 (11.6–13.2) ^a, d, e^			12.9 (11.9–13.9) ^c, e^	< 0.001^**^
Platelet count, < 100 × 10^9^ /L	157 (8.6) ^a^	3 (2.5) ^a^	27 (6.5)			4 (6.3)	0.057^***^
Fibrinogen, g/L	2.7 (2.2–3.4)	2.8 (2.3–3.4)	2.7 (2.3–3.4)			2.6 (2.1–3.4)	0.561^**^
Alpha-fetoprotein, > 400 ng/ml	692 (38.0) ^a, b^	28 (23.1) ^a, c^	133 (32.0) ^b^			28 (43.8) ^c^	< 0.001^***^
Child-Pugh grade B	170 (9.3) ^a^	5 (4.1)	23 (5.5) ^a^			5 (7.8)	0.025^***^
Barcelona Clinic Liver Cancer stage							
0/A	1083 (59.4) ^a, b^	96 (79.3) ^a, c, d^	270 (65.1) ^b, c, e^			31 (48.4) ^d, e^	< 0.001^***^
B	376 (20.6) ^a^	16 (13.2) ^a, b^	92 (22.2) ^b^			12 (18.8)	
C	363 (19.9) ^a, b, c^	9 (7.4) ^a, d^	53 (12.8) ^b, e^			21 (32.8) ^c, d, e^	
Tumor size, > 5 cm	997 (54.7) ^a, b, c^	53 (43.8) ^a^	182 (43.9) ^b^			24 (37.5) ^c^	< 0.001^***^
Multinodular tumor	433 (23.8) ^a^	10 (8.3) ^a, b, c^	97 (23.4) ^b^			21 (32.8) ^c^	< 0.001^***^
Microvascular invasion	848 (46.5) ^a, b^	42 (34.7) ^a^	151 (36.4) ^b^			24 (37.5)	< 0.001^***^

Values are expressed as number (per cent) for categorical variables, as median (interquartile range) for continuous data in case of skewed distribution and mean ± SD Continuous data showing a normal distribution. ^*^Kruskal–Wallis test was used, and Bonferroni correction was made for post hoc pairwise comparisons; ^**^One-way ANOVA was used, and LSD was made for post-hoc test; ^***^Pearson’s chi-squared test was used; ^a, b, c, d, e, f^The same letter represents significant differences between groups (*p* < 0.05); Level of significance was set at 0.05 in all statistical tests

CHB, patients with hepatocellular carcinoma associated with chronic hepatitis B virus infection but not metabolic dysfunction-related fatty liver disease (MAFLD) or chronic hepatitis C virus infection; CHB/MAFLD, HCC associated with concurrent MAFLD and chronic hepatitis B virus infection, but not chronic hepatitis C virus infection; MAFLD, HCC associated with MAFLD but not chronic infection with hepatitis B or C virus; HCV, patients with HCC associated with chronic hepatitis C virus infection in the presence or absence of chronic hepatitis B virus infection and presence or absence of MAFLD.

Compared to patients chronically infected with hepatitis virus without MAFLD, those with MAFLD but no chronic infection were significantly older and had significantly higher body mass index, systolic and diastolic blood pressure, fasting blood glucose, triglycerides and prevalence of type 2 diabetes and early-stage HCC. Conversely, those with MAFLD showed significantly shorter prothrombin time and lower prevalence of alpha-fetoprotein > 400 ng/ml, liver cirrhosis, Child-Pugh grade B, multinodular tumors and current smoker (Table 1).

Compared to patients who had MAFLD but no chronic hepatitis virus infection and patients chronically infected with hepatitis C virus with or without MAFLD, patients chronically infected with hepatitis B virus without MAFLD showed significantly higher prevalence of tumor size > 5 cm or microvascular invasion. Compared to patients with MAFLD but no chronic hepatitis virus infection and patients chronically infected with hepatitis B virus without MAFLD, those chronically infected with hepatitis C virus with or without MAFLD showed significantly higher levels of alanine aminotransferase and aspartate aminotransferase, but significantly lower levels of albumin and prealbumin. The four groups did not differ significantly from one another in sex distribution, or alcohol use, prevalence of platelet count < 100 × 10^9^ /L, prevalence of Child-Pugh grade B, or levels of total bilirubin, urea nitrogen or fibrinogen (Table 1).

**Fig. 2:**
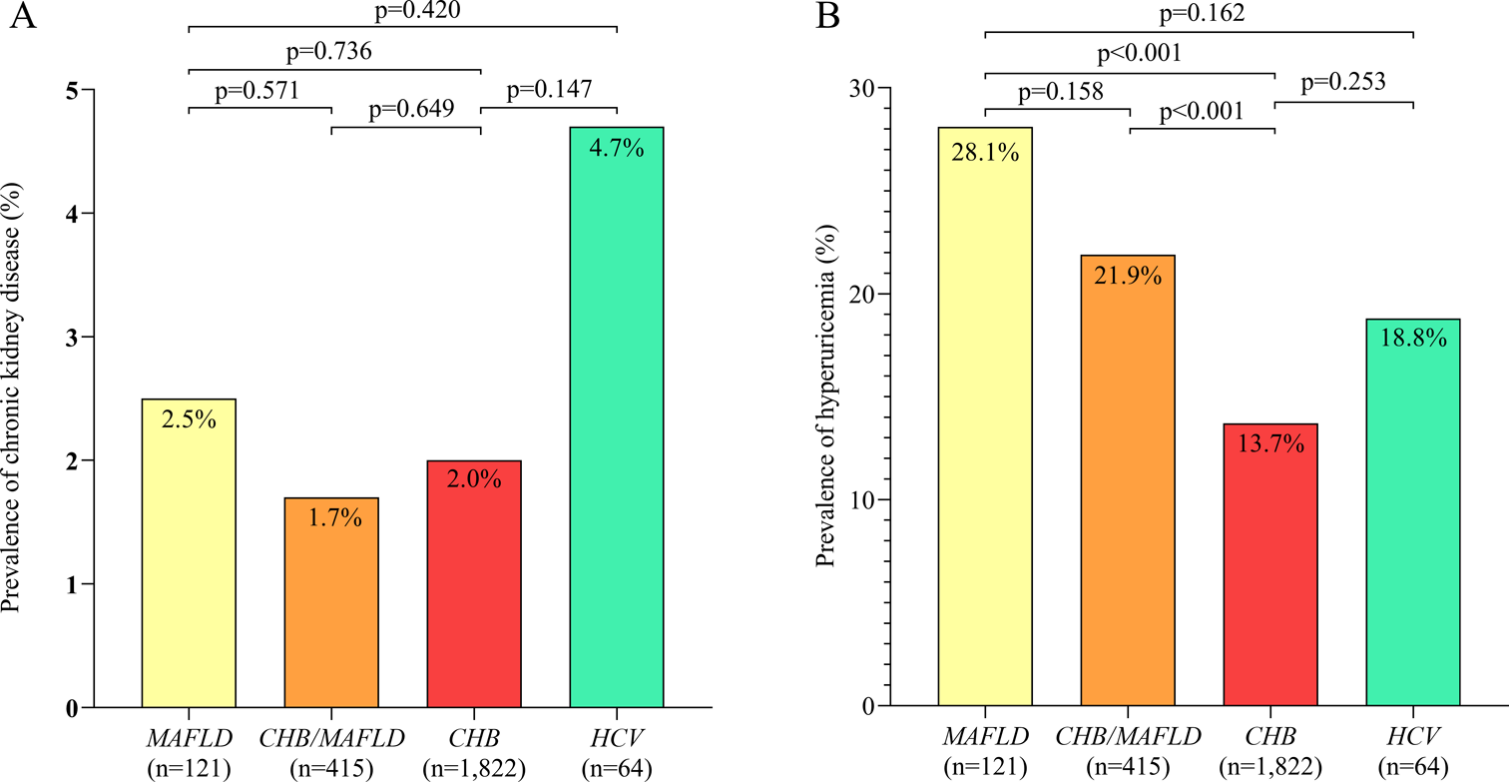
Prevalence of **(A)** metabolic syndrome, **(B)** hypertension, **(C)** type 2 diabetes, **(D)** overweight and **(E)** hypertriglyceridemia. among patients with hepatocellular carcinoma associated with chronic HBV infection but not MAFLD or chronic HCV infection (“*CHB*”); HCC associated with MAFLD but not chronic infection with HBV or HCV (“*MAFLD*”); HCC associated with concurrent MAFLD and chronic HBV infection, but not chronic HCV infection (“*CHB/MAFLD*”); or HCC associated with chronic HCV infection in the presence or absence of chronic HBV infection and the presence or absence of MAFLD (“*HCV*”). HBV, hepatitis B virus; HCV, hepatitis C virus; MAFLD, metabolic dysfunction-associated fatty liver disease

Among the different HCC etiologies, MAFLD in the absence of chronic hepatitis virus infection was associated with the highest prevalence of MetS, hypertension and type 2 diabetes, while chronic infection with hepatitis B virus in the absence of MAFLD was associated with the lowest prevalence of hypertension, type 2 diabetes, abdominal obesity, and hypertriglyceridemia (Fig. 2A-E). Among all patients with MAFLD, prevalence of abdominal obesity, type 2 diabetes or hypertriglyceridemia did not differ significantly between those chronically infected with hepatitis B virus or not.

**Fig. 3:**
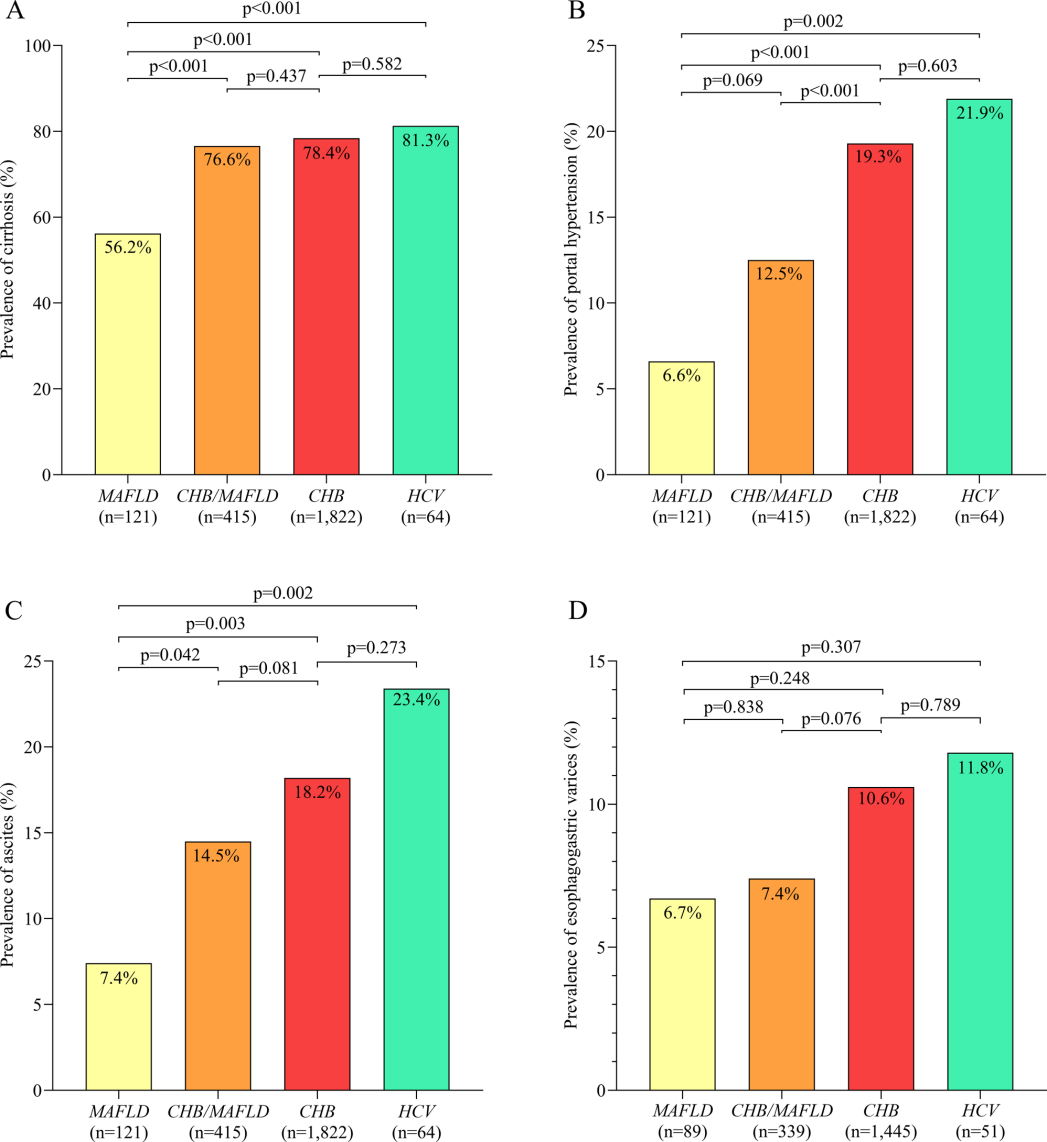
Prevalence of **(A)** history of cardiovascular disease (CVD), **(B)** high risk of ASCVD, **(C)** premature atrial beats, **(D)** T-wave alterations, and **(E)** abnormal levels of myocardial enzymes. among patients with hepatocellular carcinoma associated with chronic HBV infection but not MAFLD or chronic HCV infection (“*CHB*”); HCC associated with MAFLD but not chronic infection with HBV or HCV (“*MAFLD*”); HCC associated with concurrent MAFLD and chronic HBV infection, but not chronic HCV infection (“*CHB/MAFLD*”); or HCC associated with chronic HCV infection in the presence or absence of chronic HBV infection and the presence or absence of MAFLD (“*HCV*”). HBV, hepatitis B virus; HCV, hepatitis C virus; MAFLD, metabolic dysfunction-associated fatty liver disease; ASCVD, arteriosclerotic cardiovascular disease

Compared to patients chronically infected with hepatitis B virus without MAFLD, those with MAFLD but not chronically infected with hepatitis B or C virus showed significantly higher prevalence of T-wave alterations and history of cardiovascular disease, as well as higher risk of developing atherosclerotic cardiovascular disease within the next 10 years. Compared to patients who were chronically infected with hepatitis B virus and who had MAFLD, those with MAFLD without chronic infection showed significantly higher prevalence of a history of cardiovascular disease. Compared to patients chronically infected with hepatitis C virus with or without MAFLD, those with MAFLD but without chronic infection showed significantly higher prevalence of T-wave alterations (Fig. 3A-E).

**Fig. 4:**
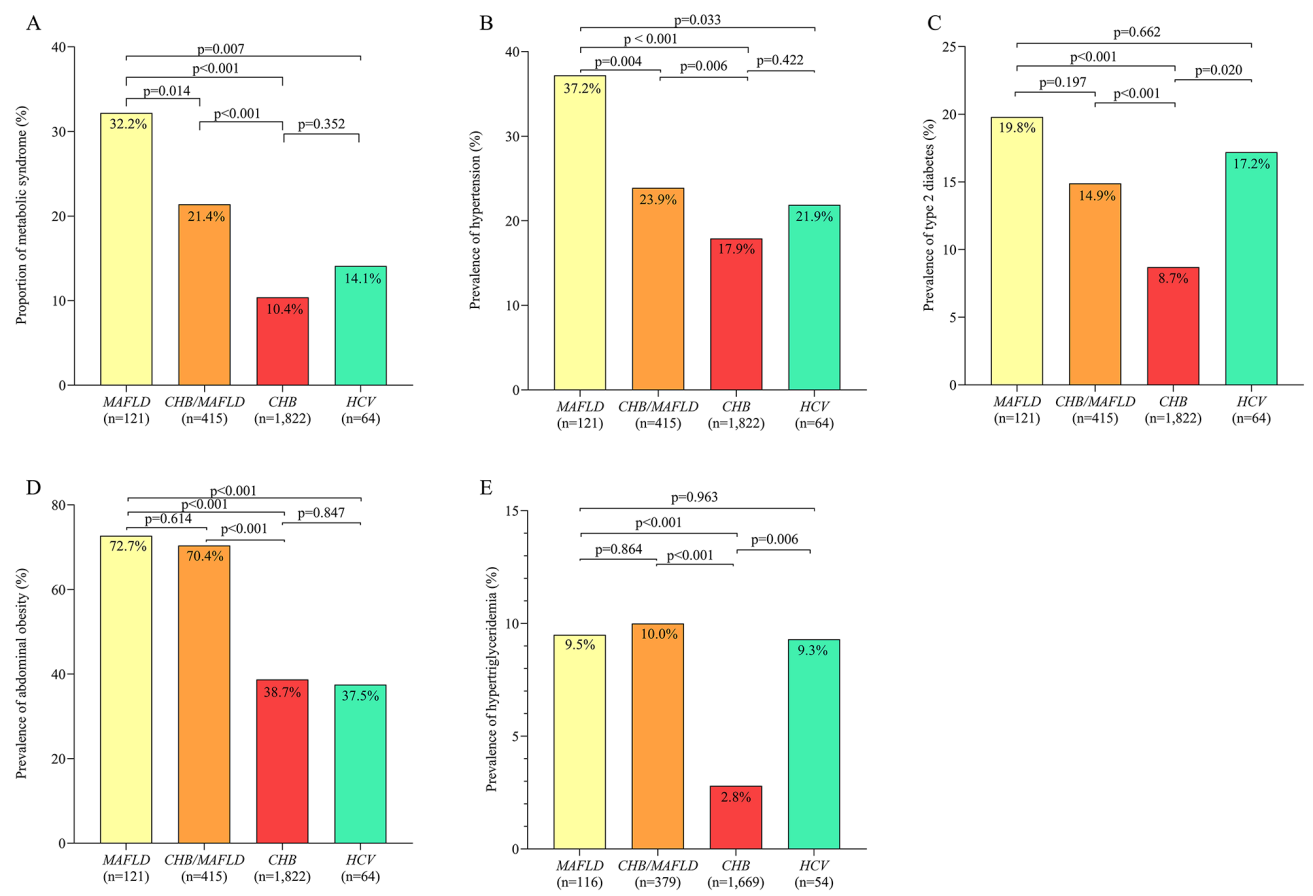
Prevalence of **(A)** chronic kidney disease (CKD), and **(B)** hyperuricemia. among patients with hepatocellular carcinoma associated with chronic HBV infection but not MAFLD or chronic HCV infection (“*CHB*”); HCC associated with MAFLD but not chronic infection with HBV or HCV (“*MAFLD*”); HCC associated with concurrent MAFLD and chronic HBV infection, but not chronic HCV infection (“*CHB/MAFLD*”); or HCC associated with chronic HCV infection in the presence or absence of chronic HBV infection and the presence or absence of MAFLD (“*HCV*”). HBV, hepatitis B virus; HCV, hepatitis C virus; MAFLD, metabolic dysfunction-associated fatty liver disease

All four groups showed similar prevalence of premature atrial beats and of abnormal levels of myocardial enzymes. They also showed similar prevalence of chronic kidney disease, even if hyperuricemia was significantly more prevalent among patients with MAFLD in the absence or presence of chronic hepatitis B virus infection than among those chronically infected with the same virus but without MAFLD (Fig. 4A-B).

**Fig. 5:**
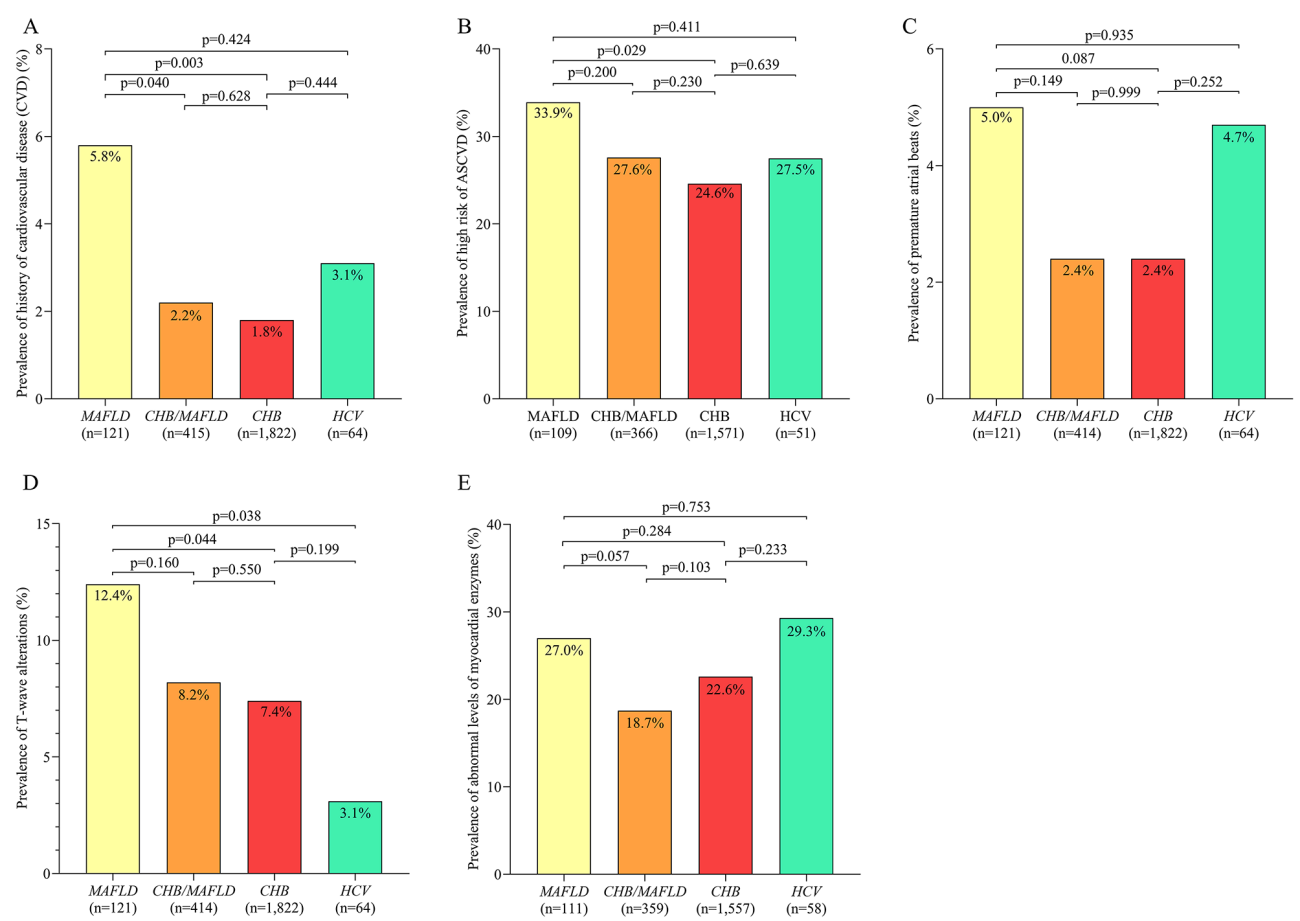
Prevalence of **(A)** cirrhosis, **(B)** portal hypertension, **(C)** ascites, and **(D)** esophagogastric varices. among patients with hepatocellular carcinoma associated with chronic HBV infection but not MAFLD or chronic HCV infection (“*CHB*”); HCC associated with MAFLD but not chronic infection with HBV or HCV (“*MAFLD*”); HCC associated with concurrent MAFLD and chronic HBV infection, but not chronic HCV infection (“*CHB/MAFLD*”); or HCC associated with chronic HCV infection in the presence or absence of chronic HBV infection and the presence or absence of MAFLD (“*HCV*”). HBV, hepatitis B virus; HCV, hepatitis C virus; MAFLD, metabolic dysfunction-associated fatty liver disease

Among the different HCC etiologies, MAFLD in the absence of chronic hepatitis virus infection was associated with the lowest prevalence of cirrhosis. It was also associated with significantly lower prevalence of portal hypertension and ascites than chronic infection with hepatitis B virus in the absence of MALFD or chronic infection with hepatitis C virus in the presence or absence of MALFD (Fig. 5A-C). Among patients chronically infected with hepatitis B virus, those who also had MAFLD showed significantly lower prevalence of portal hypertension. Among patients with MAFLD, those also chronically infected with hepatitis B virus showed significantly higher prevalence of ascites.

Among the different HCC etiologies, chronic infection with hepatitis C virus in the absence or presence of MAFLD was associated with the highest prevalence of cirrhosis, portal hypertension, ascites, and esophagogastric varices (Fig. 5A-D).

## Discussion

The inconsistent prognosis of HCC patients with different etiologies may be related to their preoperative presentation of different comorbidities. Our analysis suggests that HCC related to MAFLD frequently involves metabolic syndrome and/or comorbidities affecting the heart or kidney, but less severe liver disease than HCC related to chronic infection with hepatitis B virus. HCC related to chronic infection with hepatitis C virus may often involve severe metabolic syndrome and severe liver disease.

In our study population, HCC related to MAFLD was associated with older age, higher body mass index, higher systolic and diastolic blood pressure, higher triglycerides and more metabolic comorbidities than HCC of other etiologies. At the same time, HCC related to MAFLD was associated with lower prevalence of cirrhosis, alpha-fetoprotein concentration > 400 ng/mL, tumor size > 5 cm, multinodular tumors, advanced tumors, or microvascular invasion than HCC of other etiologies. However, HCC related to CHB is often found to be multiple, large nodular, or advanced tumors. These findings are consistent with comparisons of HCC associated with MAFLD or HCC of other etiologies [18–21]. These findings imply that patients with chronic hepatitis virus infection may be more difficult to detect early than HCC related to MAFLD. Active surveillance measures should be implemented for patients with chronic hepatitis virus infection judged to be at high risk of HCC [22, 23]. HCC patients chronically infected with hepatitis B virus, for their part, should be carefully monitored and given antiviral therapy after hepatectomy in order to reduce risk of recurrence [24, 25].

Given the association between MAFLD and higher risk of cirrhosis observed in a retrospective cohort study from China [26], and given reports that a growing percentage of cirrhosis cases in coming decades will occur in individuals with non-alcoholic fatty liver disease [27], we recommend careful assessment of comorbidities and liver function in HCC patients before hepatectomy. This may be particularly important for patients chronically infected with hepatitis virus, since those patients in our study showed higher prevalence of cirrhosis, portal hypertension, and ascites than patients with MAFLD but without chronic infection.

The relatively high prevalence of hypertension among our patients with MAFLD but without chronic hepatitis virus infection (37.2%) is consistent with hypertension as a risk factor for MAFLD [28]. The relatively high prevalence of hypertension among patients chronically infected with hepatitis C virus with or without MAFLD (25.0%) is consistent with observations linking such infection to higher risk of hypertension [29].

Prevalence of type 2 diabetes was higher in our patients with MAFLD than in those without it who were chronically infected with hepatitis B virus, similar to a study involving 4.6-year follow-up that linked MAFLD to type 2 diabetes [30, 31]. Indeed, among our patients chronically infected with hepatitis B virus, prevalence of type 2 diabetes was higher in those who also had MAFLD. Our observation that type 2 diabetes prevalence was higher among patients chronically infected with hepatitis C virus, with or without MAFLD, than among patients chronically infected with hepatitis B virus without MAFLD may reflect the demonstrated link between infection with hepatitis C virus and greater risk of insulin resistance and diabetes [32, 33].

In our sample, HCC related to chronic infection with hepatitis B virus in the absence of MAFLD was associated with lower prevalence of hypertriglyceridemia than HCC of other etiologies [34, 35]. Therefore, it is imperative to regularly monitor the blood lipid levels of individuals diagnosed with MAFLD and to intervene swiftly to resolve hypertriglyceridemia.

A smaller proportion of our HCC patients who had MAFLD but no chronic infection with hepatitis virus had a history of cardiovascular disease than in a similar study of patients from 21 Chinese hospitals (5.8 vs. 12.7%) [36]; nevertheless, the prevalence in our study was significantly higher than the prevalence among those without MAFLD who were chronically infected with hepatitis B virus. Similarly, risk of developing arteriosclerotic cardiovascular disease was higher among our patients with MAFLD than among those chronically infected with hepatitis B virus without MAFLD, consistent with an analysis of patients from South Korea [37]. This agrees with the reported link between MAFLD and greater risk of cardiovascular disease [38, 39].

Our results suggest that HCC patients with MAFLD (32.2%) are more likely to have metabolic syndrome than HCC patients chronically infected with hepatitis B or C virus. Whether MAFLD is a cause or consequence of metabolic syndrome is unclear [40, 41]. Whatever the case, similar lifestyle and diet modifications can reduce risk of both in HCC patients. Reducing risk of MAFLD may, in turn, reduce risk of cancer progression and cardiac complications: more advanced liver fibrosis in patients with non-alcoholic fatty liver disease has been linked to greater risk of cardiac symptoms and abnormal electrocardiographic parameters [42], and we found that the prevalence of T-wave alterations was higher among our patients with MAFLD without chronic hepatitis virus infection than among our patients chronically infected with hepatitis B virus without MAFLD.

The four etiologies of HCC in our study were associated with similar prevalence of premature atrial beats and abnormal levels of myocardial enzymes. In a retrospective study from the UK [43], fatty liver disease was strongly associated with an increased risk of supraventricular and ventricular arrhythmias in patients with type 2 diabetes. A study of patients in France associated cirrhosis with elevated cardiac troponin I [44]. The difference may be because metabolic syndrome and cirrhosis imaging cardiometabolic abnormalities, which should be explored in future work.

Our patient population showed a relatively low prevalence of chronic kidney disease—e.g. lower than a similar study of patients in Germany [45] —and the prevalence was slightly higher in the subset of patients chronically infected with hepatitis C virus. This is consistent with a link between such infection and higher risk of chronic kidney disease [46]. For this reason, antiviral therapy is recommended for HCC patients who are chronically infected with hepatitis C virus and who have chronic kidney disease [47].

Our observation of significantly higher prevalence of hyperuricaemia among patients with MAFLD and without chronic hepatitis virus infection than among patients chronically infected with hepatitis B virus and without MAFLD is consistent with previous studies of patients from other parts of China [48–50]. One possibility is that hyperuricaemia contributes to MAFLD, which should be explored in future work. If so, mitigating or preventing hyperuricaemia might reduce the risk of MAFLD.

### Limitations

Our findings should be interpreted with caution in light of the retrospective, single-center design of our study and our reliance on self-report for several variables, all of which may increase risk of bias. While our sample contained a relatively large number of patients chronically infected with hepatitis B virus, the numbers of those chronically infected with hepatitis C virus or diagnosed with MALFD without chronic infection were relatively small. We cannot exclude that a small number of patients with MAFLD were missed in our study because of the difficulty in detecting mild hepatic steatosis through imaging or histopathology of surgical biopsies.

## Conclusion

This retrospective study suggests that HCC associated with MAFLD tends to involve background of less severe liver disease but higher likelihood of metabolic syndrome or comorbidities affecting the heart or kidneys than HCC associated with chronic hepatitis B virus infection in the presence or absence of MAFLD. HCC associated with chronic hepatitis C virus infection, in contrast, is more likely to involve cirrhosis. Our findings should be verified and extended in larger, multi-center samples.

## Data Availability

The data that support the findings of this study are available on request from the corresponding author, Z-J-H，upon reasonable request.
